# Donor Site Morbidity of Patients Receiving Vertical Rectus Abdominis Myocutaneous Flap for Perineal, Vaginal or Inguinal Reconstruction

**DOI:** 10.1007/s00268-020-05788-5

**Published:** 2020-09-29

**Authors:** Vera S. Schellerer, Lenka Bartholomé, Melanie C. Langheinrich, Robert Grützmann, Raymund E. Horch, Susanne Merkel, Klaus Weber

**Affiliations:** 1 Department of Surgery, University Medical Center Erlangen, Friedrich-Alexander-University Erlangen-Nürnberg, Krankenhausstrasse 12, 91054 Erlangen, Germany; 2 Department of Plastic Surgery, University Medical Center Erlangen, Friedrich-Alexander-University Erlangen-Nürnberg, Krankenhausstrasse 12, 91054 Erlangen, Germany

## Abstract

**Background:**

Management of donor site closure after harvesting a vertical rectus abdominis myocutaneous (VRAM) flap is discussed heterogeneously in the literature. We aim to analyze the postoperative complications of the donor site depending on the closure technique.

**Methods:**

During a 12-year period (2003–2015), 192 patients in our department received transpelvic VRAM flap reconstruction. Prospectively collected data were analyzed retrospectively.

**Results:**

182 patients received a VRAM flap reconstruction for malignant, 10 patients for benign disease. The median age of patients was 62 years. 117 patients (61%) received a reconstruction of donor site by Vypro® mesh, 46 patients (24%) by Vicryl® mesh, 23 patients (12%) by direct closure and 6 patients (3%) by combination of different meshes. 32 patients (17%) developed in total 34 postoperative complications at the donor site. 22 complications (11%) were treated conservatively, 12 (6%) surgically. 17 patients (9%) developed incisional hernia during follow-up, with highest incidence in the Vicryl® group (*n* = 8; 17%) and lowest in the Vypro® group (*n* = 7; 6%). Postoperative parastomal hernias were found in 30 patients (16%) including three patients with simultaneous hernia around an urostomy and a colostomy. The highest incidence of parastomal hernia was found in patients receiving primary closure of the donor site (*n* = 6; 26%), the lowest incidence in the Vypro® group (*n* = 16; 14%).

**Conclusion:**

The use of Vypro® mesh for donor site closure appears to be associated with a low postoperative incidence of complications and can therefore be recommended as a preferred technique.

## Introduction

Over the last decades, main advances in treatment of malignancies have been made [1]. Surgical techniques became more radical and multimodal therapy concepts optimized the treatment of advanced malignant diseases. In advanced pelvic malignancies, large perineal voids are generated after radical operation. The tissue is altered after neoadjuvant treatment, wound healing is impaired. Often patients suffer from large perineal defects with persisting secretion or infection causing prolonged wound healing resulting in a reduced quality of life and a prolonged return to normal activity [2–6]. Furthermore, perineal herniation or perineal sinus formation may occur [7]. To avoid these problems, musculocutaneous flaps are used to fill the resulting pelvic void [8, 9]. The non-irradiated tissue of a well-vascularized flap bridges the altered irradiated wound edges and an uncomplicated wound healing is established [4]. In the literature, different flaps have been described for perineal reconstruction such as gracilis, posterior thigh, greater omentum and vertical rectus abdominis musculocutaneous flaps (VRAM flap) [8, 10–15].

In our department, patients receiving an abdominoperineal resection (APR), an extra-levator abdominoperineal excision (ELAPE) or patients who are suffering from large perineal or inguinal defects were evaluated for VRAM flap reconstruction. On the one hand, this flap is a reliable and safe method for immediate pelvic reconstruction in these patients [16], but on the other hand, a sufficient perineal closure bears the risk of a weakening of the abdominal wall and possible postoperative complications at the donor site. There are no final recommendations in current guidelines for the closure of the abdominal donor site following a VRAM flap lifting. One possibility is the primary closure between the ipsilateral external oblique muscle and the contralateral rectus abdominis muscle, which always results in high tension of the tissue. Component separation techniques [8, 17] as well as the use of different meshes for reinforcing the abdominal wall are described [18, 19]. All these techniques are discussed in the literature with heterogeneous results.

## Patients and methods

This retrospective observational cohort study includes all consecutive patients who underwent closure of perineal and inguinal defects by vertical rectus abdominis musculocutaneous (VRAM) flap reconstruction at the Department of Surgery, University Hospital Erlangen, Erlangen, Germany, between January 2003 and September 2015. The aim of the present study was to evaluate postoperative donor site morbidity and the incidence of incisional as well as parastomal hernia after VRAM flap reconstruction for inguinal, vaginal or perineal defects.

### Surgical technique

VRAM-flap harvest is described in detail by Horch et al. [16, 20]. In brief, the size and shape of the flap is determined intraoperatively depending on the defect to be covered. Once the size of the skin island has been determined, cylindrical preparation of the anterior fascia of the rectus abdominis muscle is performed. The perforators penetrating the deep fascia were dissected. Doing this way, the fascial defect of the abdominal wall can be kept as small as possible. After harvesting the VRAM flap, the anterior rectus fascia is preserved below the arcuate line of the rectus sheath. This enables a primary closure of the abdominal wall caudally. For protection of the epigastric vessels, the inferior insertion of the muscle at the pubic ramus is not disinserted. After the VRAM flap is finally transferred, the posterior rectus sheath is closed with absorbable sutures primarily. A gap is left above the symphysis to preserve the base of the VRAM flap and the vessels supplying the flap. In this area, the preserved anterior rectus sheath is also primarily closed. At the level of the resulting muscular defect above the arcuate line, this defect of the anterior rectus sheath is reconstructed by the following four techniques: primary closure of the abdominal wall, enforcement of abdominal fascia by Vypro® mesh (Ethicon, Johnson & Johnson, Norderstedt, Germany), Vicryl® mesh (Ethicon) or different techniques. The Vypro® mesh consists of absorbable and non-absorbable multifilament yarn. The absorbable polyglactin yarn is a copolymer of 90% glycolide and 10% lactide. The non-absorbable yarn consists of polypropylene. The Vicryl® mesh consists of fully absorbable polyglactin. Primary closure means the conventional abdominal wound closure without any mesh implantation. Different techniques include the combination of a Vypro® and a Vicryl® mesh implantation in two patients and the use of polypropylene-meshes in four patients. After subcutaneous closure, two suction drains are inserted and the skin is closed with a skin stapler.

Postoperative complications were classified into minor complications requiring non-surgical treatment, and major complications with the need for surgery, regardless of other possible synchronous complications.

The American Society of Anesthesiologists (ASA) score, body mass index (BMI), and nicotine and alcohol abuse were evaluated to assess the general health status of the patients. Medical records were analyzed for patient demographics, intraoperative data and postoperative outcome. Patients were followed until death or 30th December 2015.

### Statistical analysis

All statistical analyses were performed using the statistical software package SPSS® version 21.0 (IBM, Amonk, New York, USA). The chi-square test and Fisher’s exact test were used for comparison of categorical data; the Mann–Whitney U test was utilized to compare continuous data. A *p* value <0.05 was determined statistically significant.

## Results

### Patient demographics

The VRAM flap was used to close perineal, vaginal and other pelvic defects in 188 patients (98%), inguinal defects in 3 (1.5%) and intraabdominal defects in 1 (0.5%). The perineal defects occurred mainly following APR or ELAPE, the inguinal defects after extensive groin resection of sarcoma or squamous cell carcinoma. In one patient, the VRAM flap was used to fill an intraabdominal abscess cavity following a multivisceral resection (resection of the os ileum, small and large intestine) of recurrent colon cancer. In 182 patients (95%) malignant and in 10 patients (5%) benign underlying diseases were present.

In 163 patients, the defect of the donor site was on the right side and in 29 patients on the left side of the abdominal wall. Preferably, the terminal colostomy is placed on the left side through the abdominal wall, accordingly the VRAM flap is lifted from the right side. The main reasons for harvesting a left-sided VRAM flap were previous operations on the right side of the abdomen and insufficient right-sided epigastric vessels or perforator vessels. Patients’ characteristics are presented in Table 1.

**Table 1:** Patients’ characteristics (*n* = 192)

	*n* (%)
Age (years)	
Median	62
Range	29–89
Sex	
Male	121 (63)
Female	71 (37)
ASA^a^	
1–2	128 (67)
3–4	63 (33)
BMI (kg/m^2^)^b^	
Mean	25.6
Range	16–44
Alcohol (frequent consumption active or prior)^c^	31 (16)
Nicotine (active smoker)^b^	33 (17)
*Perineal defects*	189 (98.4)
Malignant diseases	162 (85.7)
Rectal carcinoma	133
Anal carcinoma	14
Anorectal fistula carcinomas	3
Squamous cell carcinoma perineal, gluteal	2
Cervix carcinoma	2
Sigmoid carcinoma	2
Urothelial carcinoma	2
Vulvar carcinoma	1
Rectal gastrointestinal stroma tumor	1
Malignant melanoma of the anal canal	1
Presacral sarcoma	1
Benign diseases	26 (14.3)
Morbus crohn	6
Colitis ulcerosa	1
Familial adenomatous polyposis	1
Chondroma	1
Vaginal perforation	1
Chronic anal fistulas following surgery for malignancies	16
*Inguinal defects*	3 (1.6)
Alveolar sarcoma inguinal	1
Liposarcoma inguinal	1
Squamous cell carcinoma inguinal	1

*ASA* American Society of Anesthesiologists Score

^a^1 patient unknown

^b^3 patients unknown

^c^7 patients unknown

### Reconstruction techniques of donor site

The abdominal wall reconstruction techniques changed at the beginning of the study. Therefore, the patients were divided into the following groups: closure of the donor-site with Vypro® meshes (I), Vicryl® meshes (II), primary closure (III), a combination of meshes (IV). Patients’ characteristics within the different groups of closure techniques are presented in Table 2. The groups did not differ in gender, age, BMI, ASA score, nicotine and alcohol consumption, diabetes mellitus and intake of immunosuppressive medications.

**Table 2:** Reconstruction techniques and patients characteristics of 192 patients

	Vypro®mesh	Vicryl®mesh	Primary closure	Different techniques/ combinations	*p* value
	117 (60.9%)	46 (24.0%)	23 (12.0%)	6 (3.1%)	
Perineal/Inguinal defects					
Malignant disease	112	45	20	5	0.111
Benign disease	5	1	3	1	
Age (years)					
Median	63.5	63	64	61	0.645
Range	29–89	37–84	36–79	36–67	
Sex					0.458
Male	76	30	11	4	
Female	41	16	12	2	
ASA^a^					
I–II	82 (70%)	30 (65%)	13 (57%)	3 (50%)	0.635
III–IV	35 (30%)	16 (35%)	10 (43%)	2 (33%)	
BMI (kg/m^2^)^b^					
Mean	25.9	24.4	23.6	26.8	0.425
Range	16–42	17–35.5	16.0–44.5	18–32.7	
BMI ≥ 30^*b*^	21 (18.4%)	6 (13%)	3 (13%)	1 (17%)	0.879
Alcohol^c^					
Active or prior	25 (22.7%)	5 (11%)	1 (4%)	0 (0%)	0.106
Nicotin^b^					
Active	17 (15%)	10 (22%)	3 (13%)	3 (50%)	0.050
Prior	25 (23%)	0 (0%)	0 (0%)	0 (0%)	
Diabetes mellitus	14 (12%)	6 (13%)	6 (26%)	1 (17%)	0.344
Immunosuppressive medication	3 (3%)	4 (9%)	2 (9%)	1 (17%)	0.124

*ASA* American Society of Anesthesiologists Score, *BMI* body mass index

^a^1 patient unknown

^b^3 patients unknown

^c^7 patients unknown

### Early postoperative donor site complications

In total, 32 patients (17%) developed 34 complications (see Table 3). Donor site complications were divided into those with need for conservative treatment and those with need for surgical treatment. 22 complications in 21 patients were treated conservatively. 10 patients had seromas and 8 patients wound infections, one patient suffered from seroma and surgical site infection, both complications were treated conservatively. 12 patients with 12 complications on the donor site needed surgical treatment, for fascial dehiscence (*n * = 4), seroma (*n* = 3), wound infection (*n* = 2), hematoma (*n* = 2) and umbilical necrosis (*n*  = 1). Complications varied significantly (*p* = 0.018) between the groups. There were significantly more complications in the groups with closure of the abdominal wall with Vicryl® mesh (28%) and with combinations of meshes (50%) compared to Vypro® meshes (13.7%) and primary closure (9%) of the abdominal wall. This significant difference was also evident in conservative manageable complications in general (*p* = 0.031) and seromas (*p* = 0.025) as well as hematomas needing surgical treatment (*p* = 0.037).

**Table 3:** Postoperative complications of 192 patients, multiple answers were possible

*n*	All	Vypro® mesh	Vicryl®mesh	Primary closure	Different techniques/ combinations	*p* value
	192	117	46	23	6	–
Any complication	33 (17.2%)^a^	16 (13.7%)	13 (28%)	2 (9%)	3 (50%)	0.018
*Complications needing conservative treatment*	21 (10.9%)^b^	11 (9.4%)	9 (20%)	0	2 (33%)	0.031
Wound infection	8 (4.2%)	3 (2.6%)	5 (11%)	0	0	0.106
Seroma	10 (5.2%)	4 (3.4%)	4 (9%)	0	2 (33%)	0.025
Hematoma	2 (1.0%)	2 (1.0%)	0	0	0	1.0
Granuloma	1 (0.5%)	1 (1%)	0	0	0	1.0
Chronical pain syndrome	1 (0.5%)	1 (1%)	0	0	0	1.0
*Complications needing surgical treatment*	12 (6.3%)	5 (4.3%)	4 (9%)	2 (9%)	1 (17%)	0.234
Wound infection	2 (1.0%)	1 (0.9%)	1 (2%)	0	0	0.630
Seroma	3 (1.6%)	3 (2.6%)	0	0	0	0.731
Hematoma	2 (1.0%)	0	1 (2%)	0	1 (17%)	0.037
Fascial dehiscence	4 (2.1%)	1 (0.9%)	2 (4%)	1 (4%)	0	0.279
Umbilical necrosis	1 (0.5%)	0	0	1 (4%)	0	0.151

^a^33 patients with 34 complications

^b^21 patients with 22 complications

### Late postoperative donor site complications

During follow-up, 17 patients (9%) developed incisional hernia, including 11 patients with incisional hernia alone and six patients with incisional hernia in addition to a parastomal hernia (Table 4). The highest rate of incisional hernia was found in patients receiving a Vicryl® mesh (*n* = 8; 17%), the lowest (*n* = 7; 6%) in patients with re-enforcement of the abdominal wall by a Vypro® mesh.

**Table 4:** Late complications

	All	Vypro® mesh	Vicryl® mesh	Primary closure	Different techniques/ combinations	*p* value
All patients	192	117	46	23	6	–
Incisional hernia	17 (9%)	7 (6%)	8 (17%)	2 (9%)	0	0.129
Incisional hernia alone	11	5	6	0	0	0.137
Incisional hernia in combination with one parastomal hernia	4	2	2	0	0	0.516
Incisional hernia in combination with two parastomal hernia	2	0	0	2	0	0.022
Patients with stoma^a^	188	114	45	23	6	0.370
Parastomal hernia	30 (16%)	16 (14%)	8 (18%)	6 (26%)	0	
Colostomy	27	15	8	4	0	
Urostomy	3	1	0	2	0	

^a^4 patients without stoma: VRAM flap used for closure of inguinal defects (*n* = 3), intraabdominal defect (*n*  = 1)

For the analysis of the incidence of parastomal hernia, we excluded the four patients with VRAM flap reconstruction for inguinal (*n* = 3) and intraabdominal (*n* = 1) defects. Of the 188 patients included, 44 patients had 48 abdominal stomata prior to the VRAM flap operation, including colostomies (*n* = 28), ileostomies (*n* = 10), urostomies (*n* = 2), colostomies in combination with urostomies (*n* = 2), colostomy in combination with ileostomy (*n* = 1) and urostomy in combination with ileostomy (*n* = 1). During VRAM flap operation 139 colostomies, 3 ileostomies and 13 urostomies were generated. There were 13 patients with a transformation of a loop colostomy into a terminal colostomy and 11 patients with a transformation of an ileostomy into a colostomy. During postoperative follow-up, 30 patients developed parastomal hernias, including 4 patients with already existing stomata (without documented evidence of parastomal hernia at the time of VRAM flap surgery). All other stomata were implanted during the operation with the VRAM flap reconstruction. Because 3 patients developed a parastomal hernia on both the colostomy and the urostomy, a total of 33 parastomal hernias occurred. In total, 30 out of 188 patients (16%) developed 33 parastomal hernias. The highest incidence rate was found in patients with primary closure of the abdominal wall (*n* = 6; 26%) and the lowest in patients with closure of the abdominal defect with Vypro® mesh (*n* = 16; 14%, Table 4).

**Fig. 1:**
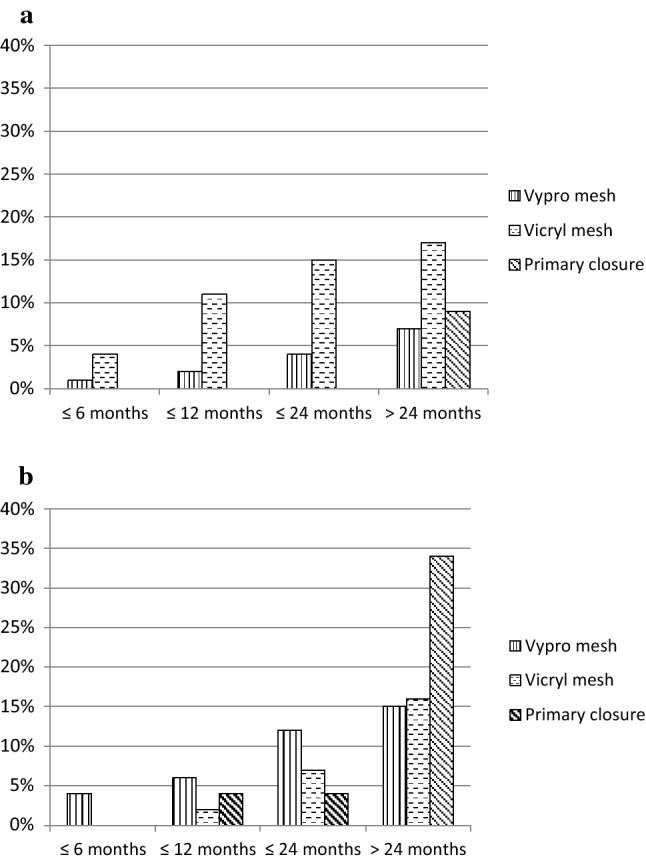
Cumulative incidence of incisional (**a**) and parastomal (**b**) hernia within two years after operation

During follow-up, the cumulative incidences of incisional and parastomal hernia increased for all groups (Fig. 1).

## Discussion

The interdisciplinary approach of reconstructing major pelvic or perineal wound defects immediately during oncological surgery is very advantageous for the patient. In our center, we prefer the vertical rectus abdominis musculocutaneous (VRAM) flap to reconstruct large perineal or inguinal wounds [11, 16]. However, this procedure generates a new defect in the abdominal wall, which itself might lead to postoperative complications. Recent developments of near infrared angiography and hyperspectral analysis of skin and muscle perfusion could eventually help to optimize abdominal wall closure by defining the microcirculation of wound edges [21].

**Fig. 2:**
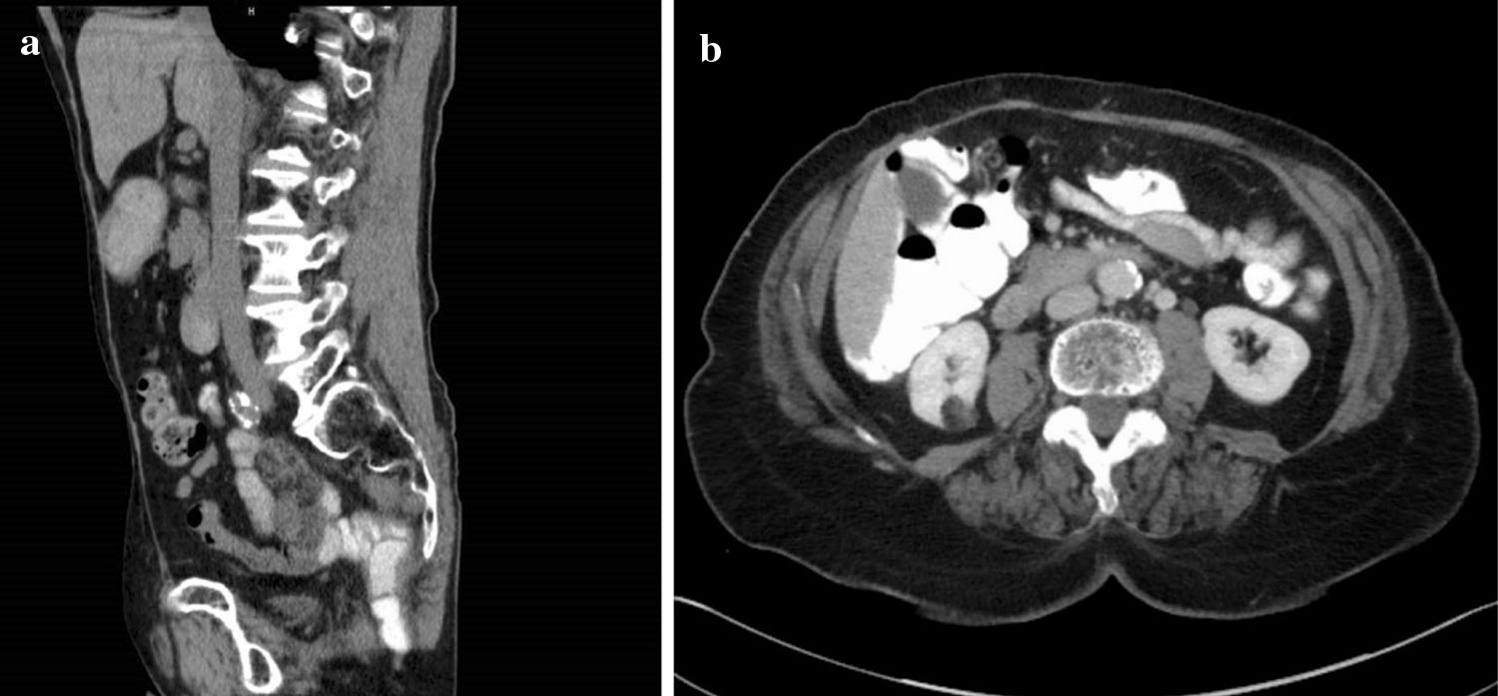
Sagittal (**a**) and axial (**b**) computed tomography scans of a stable abdominal wall 33 months after donor site closure with a doubled Vypro® mesh

The aim of this study was to analyze postoperative long- and short-term complications of the abdominal donor site of the VRAM flap and to describe a reconstruction technique that in our opinion achieves beneficial postoperative results and stability of the abdominal wall (Fig. 2). To our knowledge, our cohort of 192 patients is one of the largest cohorts of a single-center published to date. During the period of investigation, the reconstruction technique of the donor site after lifting a VRAM flap changed in our department, but study data were missing. As we recognized possible advantages of the Vypro® mesh over the other methods in this procedure, this study was initiated. The general condition of the patients (e.g., obesity, nicotine/alcohol abuse, ASA score) was analyzed in addition to evaluate a possible influence on the postoperative complication rates.

**Fig. 3:**
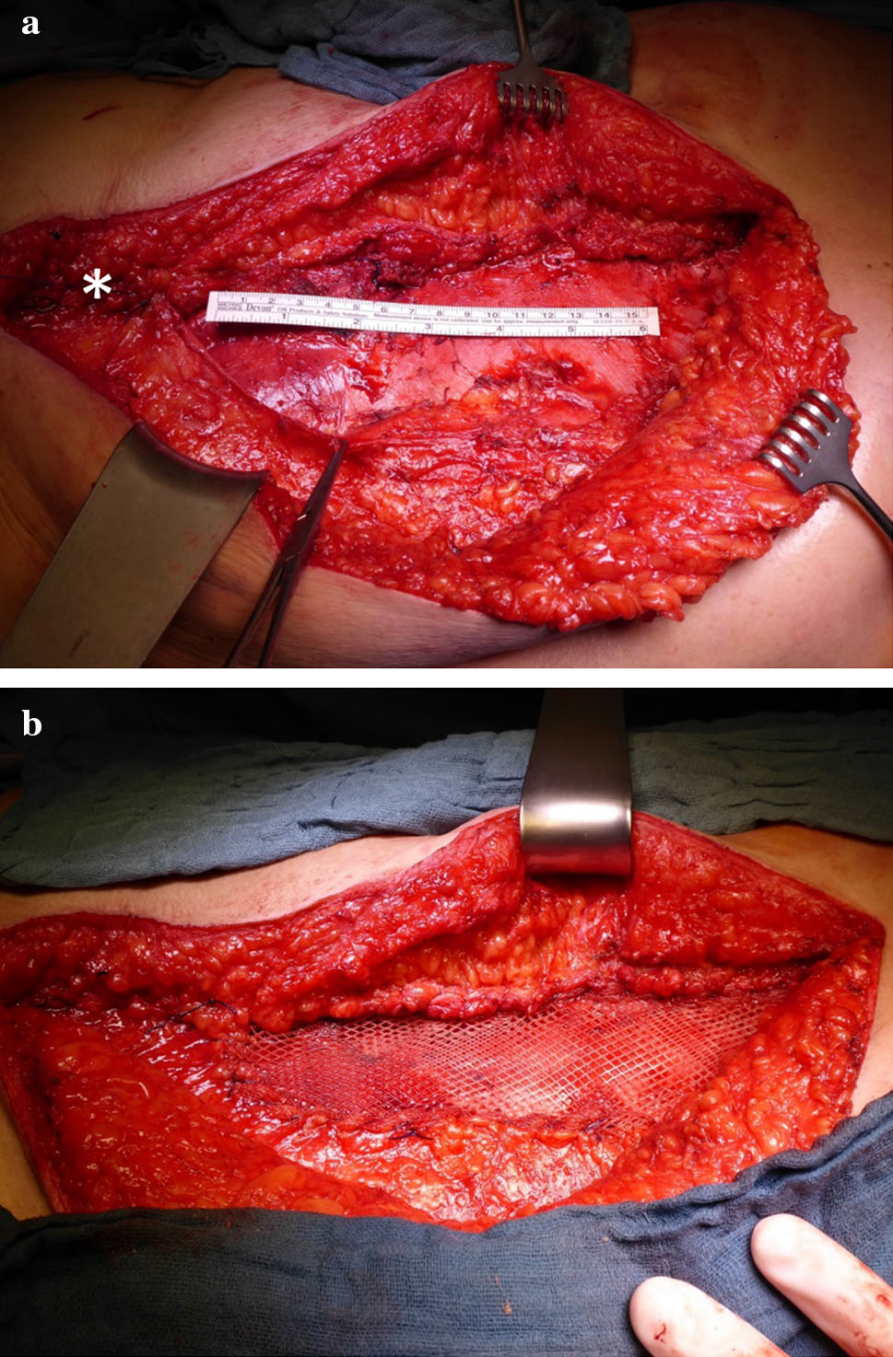
Site after lifting a left-sided vertical rectus muscle flap. The VRAM flap was lifted and transferred to the perineal wound. The posterior rectus sheet is closed by absorbable sutures. The caudal part of the wound (*) with intact left rectal muscle is closed by direct sutures between the anterior muscle sheets. The donor site defect is about 17 cm × 5 cm (**a**). Intraoperative site after reconstruction of the donor site defect with a doubled Vypro® mesh (**b**)

In the literature, many techniques exist for reconstruction of the abdominal wall following the lifting of a VRAM flap, e.g., direct closure, component-separation, biological or synthetic mesh reinforcement [17–19, 22, 23]. In our patient collective, we re-enforced the abdominal wall with different alloplastic materials (absorbable or non-absorbable meshes) or closed the abdominal wall primarily. During the last 10 years, the following standard was established: the caudal part of the wound (* in Fig. 3 a) with intact left rectal muscle is closed by direct sutures between the anterior muscle sheets (Fig. 3a). Subsequently, the defect of the anterior rectus sheath is bridged by a doubled Vypro ® mesh, whereby the mesh is inserted tightly (Fig. 3b) and sutured running with Vicryl 1 ct. The main complications on the donor site included surgical side infections, fascia dehiscence, necrosis of the skin and hernia, representing the complications described previously in the literature [8, 18, 19, 24]. Our incidence rate of 17% of postoperative complications at the donor site is comparable to the literature (23% postoperative complications by Houdek et al., 21% postoperative complications by Campbell et al., 29% postoperative complications by Butler et al., 16% postoperative complications by Sunesen et al.) [8, 18, 19, 24]. In our patients, most early complications (63%) were manageable conservatively. We noticed that direct closure of the abdominal donor site resulted in low rates of short-time postoperative complications (like seroma, wound infection). Interestingly, in patients with direct closure of the fascia, we did not find any complication treatable conservatively, but fascial dehiscence and umbilical necrosis, which needed further surgical re-intervention. Direct closure of the fascia leads to high traction of the abdominal wall and an increased risk of parastomal and incisional hernia in the future. In our study, the “primary closure group” presented with the highest incidence of parastomal (26%) and incisional hernia (9%) during follow-up. In contrast, Butler et al. demonstrated no significant difference in abdominal wall complications comparing patients with and without VRAM flap harvest. In both groups, the abdominal wall was closed by interrupted or running polypropylene sutures without prosthetic mesh and there were no differences in incidence of abdominal skin or fascial dehiscence and incisional or parastomal hernia [8]. Other studies with different techniques of closure of the abdominal wall (such as primary fascial closure, component separation, absorbable/non-absorbable meshes) found an incidence of incisional hernia between 0 and 24% and for parastomal hernia between 7 and 16% [8, 10, 17, 18, 24–29]. For parastomal hernia without prophylactic mesh reinforcement around the ostomy, incidence rates up to 50% after APR are described [7]. Also Baumann et al. demonstrated a higher risk for early and late donor site complications in case of primary fascial closure compared to component separation technique. In his study, the “primary closure group” presented a fourfold incidence rate for incisional hernia compared to the component separation group, also the incidence of parastomal hernia was slightly higher [17]. Parastomal hernia is clinically difficult to diagnose. Compared to physical examination, the detection rate by computerized tomographic evaluation is described up to 20% higher [30, 31]. Depending on the applied detection mode, there might be an underestimation of the true incidence rate and comparison of different studies might be difficult, in particular as length of follow-up also increases the detection rate [32]. In the literature, there are significantly reduced rates of parastomal hernia in case of prophylactic mesh reinforcement described [7, 33]. In our department, prophylactic reinforcement of colostomies by Vypro® mesh has been performed since 4 years. However, given the data from Butler et al. it remains unclear if harvesting a VRAM- flap from the contralateral abdominal wall does have a direct correlation with the development of parastomal hernias, or if this is independent from this reconstructive procedure [8].

Most postoperative complications were found in the Vicryl® group. 20% of patients were treated conservatively for complications like wound infections and seromas. 9% needed surgical therapy for fascial dehiscence and wound infection. Also, the incidence of incisional hernia was 18% in this group, mainly because Vicryl® absorbs within several weeks after operation and stability of the abdominal wall weakens over time after operation. We used Vicryl® mesh only in the beginning of VRAM flap reconstructions, as we were afraid of mesh infection due to bacterial contamination.

As Vicryl® absorbs within several weeks and Prolene® mesh implies a high risk of persisting infection, we introduced Vypro® mesh in our department for reinforcement of the abdominal wall. Vypro® enables sufficient long-term stability of the abdominal wall with a low risk of persisting infection when used in a contaminated surgical field. We could prove the advantages of Vypro® mesh in our study cohort as this group presented with the lowest incidences of complications with regard to surgical re-intervention. In addition, a good stability of the abdominal wall could be achieved even during follow-up (Fig. 2).

Due to the small number of patients (*n* = 6) included in the group with different mesh combinations and the resulting low significance, these results were not considered.

## Conclusion

Abdominal re-enforcement by Vypro® mesh is a reasonable procedure technique in patients with large abdominal muscle transfer. Postoperative complication rates are lower and incidences of incisional and parastomal hernias are tolerable compared to direct closure of the abdominal wall or reinforcement with fully absorbable meshes.
